# Proteomic Evidences for Rex Regulation of Metabolism in Toxin-Producing *Bacillus cereus* ATCC 14579

**DOI:** 10.1371/journal.pone.0107354

**Published:** 2014-09-12

**Authors:** Sabrina Laouami, Géremy Clair, Jean Armengaud, Catherine Duport

**Affiliations:** 1 Avignon Université/INRA, SQPOV UMR408, Avignon, France; 2 INRA, SQPOV UMR408, Avignon, France; 3 Laboratoire de Biochimie des Systèmes Perturbés, CEA Marcoule, DSV-iBEB-SBTN-LBSP, Bagnols-sur-Cèze, France; National Research Council of Italy (CNR), Italy

## Abstract

The facultative anaerobe, *Bacillus cereus*, causes diarrheal diseases in humans. Its ability to deal with oxygen availability is recognized to be critical for pathogenesis. The *B. cereus* genome comprises a gene encoding a protein with high similarities to the redox regulator, Rex, which is a central regulator of anaerobic metabolism in *Bacillus subtilis* and other Gram-positive bacteria. Here, we showed that *B. cereus rex* is monocistronic and down-regulated in the absence of oxygen. The protein encoded by *rex* is an authentic Rex transcriptional factor since its DNA binding activity depends on the NADH/NAD^+^ ratio. *Rex* deletion compromised the ability of *B. cereus* to cope with external oxidative stress under anaerobiosis while increasing *B. cereus* resistance against such stress under aerobiosis. The deletion of *rex* affects anaerobic fermentative and aerobic respiratory metabolism of *B. cereus* by decreasing and increasing, respectively, the carbon flux through the NADH-recycling lactate pathway. We compared both the cellular proteome and exoproteome of the wild-type and Δ*rex* cells using a high throughput shotgun label-free quantitation approach and identified proteins that are under control of Rex-mediated regulation. Proteomics data have been deposited to the ProteomeXchange with identifier PXD000886. The data suggest that Rex regulates both the cross-talk between metabolic pathways that produce NADH and NADPH and toxinogenesis, especially in oxic conditions.

## Introduction

*Bacillus cereus* is a Gram-positive, facultative-anaerobe, rod-shaped endospore-forming human pathogen. Most of the reported illnesses involving *B. cereus* are food-borne intoxications, classified as emetic and diarrheal syndromes [1], [2]. Diarrheal disease is due to vegetative outgrowth and secretion of various extracellular factors, including enterotoxins [3]. The most extensively studied diarrheal enterotoxins are hemolysin BL (Hbl), nonhemolytic enterotoxin (Nhe), and cytotoxin K (CytK) [4], [5]. These enterotoxins are secreted via the Sec translocation pathway [6]. Although Hbl, Nhe and CytK are currently considered as the etiologic agents of diarrheal syndrome, other toxins, such as EntA, EntB and EntC, may also contribute to the pathogenicity of *B. cereus* [7], [8]. To grow and produce virulence factors in the human intestine, *B. cereus* must adapt its metabolism, and regulates its proteome [9] in response to changes in oxygen availability. Indeed, *B. cereus* encounters oxic conditions in zones adjacent to the mucosal surface [10] and anoxic condition in the intestinal lumen [11]. Changes in oxygen availability can influence the relative levels of the dinucleotide, NAD^+^ and NADH, in the cell, and such changes are sensed by the transcriptional regulator, Rex, in *B. subtilis* [12] as in other Gram-positive bacteria [13], [14], [15], [16], [17], [18], [19]. Depending on the cellular NAD^+^/NADH ratio, Rex regulators modulate the expression of genes involved in fermentative metabolism, biofilm formation, and oxidative stress [18], [20], [21]. Structural studies of Rex proteins have identified dinucleotide-binding pockets in the C-terminal domain of the protein. NADH binding in this region leads to a conformational change in the Rex homodimer, triggering a displacement of Rex from its recognition sites on DNA, and thus leading to de-repression of the downstream genes [15], [19]. A Rex homologue has been detected in the cellular proteome of *B. cereus* [9]. Furthermore, we found a canonical Rex binding motif [15] overlapping ResD and Fnr binding motifs [22], [23], [24] in the *ldhA* promoter region [25]. In *B. cereus*, ResD, Fnr and LdhA regulate both catabolism and enterotoxin production under both aerobic and anaerobic growth conditions, probably through a regulatory complex [26]. Rex could be, thus, a regulator of both catabolism and toxinogenesis in *B. cereus*. A role of Rex in toxinogenesis has not yet been reported in *B. cereus* or in any other organism.

In this study, we show that Rex from *B. cereus*, like its orthologues, is a transcriptional factor capable of interacting with DNA in an NADH/NAD^+^-responsive manner. We demonstrate that *B. cereus* Rex is a key regulator of anaerobic fermentation, aerobic respiration, resistance against external reactive oxygen species, and toxinogenesis, by modulating the cellular and extracellular proteome in an oxygen-dependent manner. This study provides the most comprehensive experimental information on proteins whose synthesis was changed in the presence of Rex. All together, our results offer new information about the metabolic events that maximize *B. cereus* growth in environments with varying oxygen conditions.

## Materials and Methods

### Bacterial strains, media, and growth conditions

*Escherichia coli* TOP 10 (Invitrogen) was used as the host for cloning experiments, and *E. coli* SCS110 (Stratagene, La Jolla, CA) was used to prepare DNA for *B. cereus* transformation. *B. cereus* ATCC 14579 [27] was used as the parent strain for the construction of the *rex* deletion mutant. *E. coli* strains were grown at 37°C, with agitation, in Luria broth (LB). *B. cereus* strains were cultured in batches (three independent cultivations per strain) at two oxygen availabilities, i.e. pO_2_ = 0% and pO_2_ = 100% [8], [9]. Each batch culture was inoculated with a subculture grown overnight at an initial optical density at 560 nm (OD_560_) equal to 0.02. For *B. cereus* cultivation, the minimal MOD medium was supplemented with 30 mM glucose as the carbon source [28]. Anaerobic and aerobic batch cultures were performed at 37°C in a 2 L bioreactor (BioFlo/CelliGen 115, New Brunswick), and the working volume was maintained at 1.8 L. The pH was kept at a controlled value of 7.2 by automatic addition of 5 M KOH. *B. cereus* growth was monitored spectrophotometrically at 560 nm and calibrated with cell dry-weight measurements as previously described [25]. *B. cereus* cells were harvested by centrifugation when they reached their maximal growth rate (µ = µ_max_) and immediately frozen until proteomic analysis. Supernatants were kept at −20°C for glucose and glucose-by-product assays and exoproteomic analysis.

### Construction of the *B. cereus* Δ*rex* mutant strain

The deletion mutant, ATCC 14579 Δ*rex*, was constructed as follows. Two DNA fragments encompassing the 5′untranslated region (UTR) and 3′UTR of *rex* (BC 0291) were generated by PCR using primer pairs, rex1F (5′-GCCATGTTAATGTTTCGATGTCT-3′) and rex2R (5′-CCCGGGATCTTTTAGCAGTGGCTTGTGG-3′, *Sma*I restriction site is underlined), and rex3F (5′-CCCGGGGTTTACTTTTTGAAAAACTATCCACAA-3′, *Sma*I restriction site is underlined) and rex4R (5′-TGCATTATGTATCGTGCTTTGG-3′), respectively. The resulting 840 bp 3′*SmaI* and 827 bp 5′*SmaI* DNA fragments were cloned into the TA cloning vector, pCR4-TOPO (Invitrogen, La Jolla, CA), generating plasmids, pCR4mut*rex*1 and pCR4mut*rex*2, respectively. The 840 bp DNA fragment encompassing the 5′UTR region of *rex* was isolated from pCR4mut*rex*1 with *Pst*I and *Sma*I, and subcloned into pCR4mut*rex*2 to generate pCR4mut*rex*3. A 1.5 kbp *Sma*I fragment containing the entire spectinomycin gene, *spc* [29], was purified from pDIA [25]. This purified fragment was ligated into *Sma*I-digested pCR4mut*rex*3. The resulting plasmid, pCR4mut*rex*4, was digested with *EcoR*I and the resulting 3067 bp 5′rexUTR-*spc*-3′rexUTR was subsequently inserted into the *EcoR*I site of pMAD [30]. The resulting plasmid was introduced into *B. cereus* cells by electroporation. The *rex* ORF was deleted and replaced with *sp*c via a double-crossover event [30]. Chromosomal allele exchanges were confirmed by PCR with oligonucleotide primers located upstream and downstream of the DNA regions used for allelic exchange. To complement the *rex* gene in *trans*, a DNA fragment of 932 bp encompassing the *rex* ORF (630 bp) and its promoter region (219 bp) was first PCR amplified using the primer pairs, rexcompF (5′-GGATCCCGTTCGAAAGCGCGTTTACTTG-3′; the *BamH*I restriction site is underlined) and rexcompR (5′-GAGCTCGATTTTAATTTGGCACTTCGCC-3′; the *Sac*I restriction site is underlined), and then cloned into the pCRXL-TOPO plasmid (Invitrogen). The PCR fragment was then cut with *BamH*I and *Sac*I and ligated into pHT304 [31], digested with the same restriction enzymes. The integrity of the recombinant vector (pHT304*rex*) insert was verified by sequencing.

### Phenotypic characterization of *B. cereus* Δ*rex* using the API-50CHB testsystem

The carbohydrate metabolism of the wild-type strain (WT) transformed or not with pHT304, Δ*rex* mutant transformed or not with pHT304 and Δ*rex* mutant transformed with pHT304*rex* was examined using API 50 CHB strips (BioMérieux SA, France). The results showed that WT and WT(pHT304) did not ferment turanose. In contrast, Δ*rex* and Δ*rex* (pHT304) showed a typical positive reaction in turanose test fermentation. Transformation of Δ*rex* mutant with pHT304*rex* inhibited the capacity of Δ*rex* to ferment turanose.

### Measurement of glucose and by-product concentrations

Enzymatic test kits from Diffchamb (Lyon, France), R-Biopharm (Saint-Didier au Mont-d’Or, France), and Roche (Meylan, France) were used to analyze the glucose, lactate, ethanol, formate, acetate, and succinate concentrations in the supernatants of 4 mL cell cultures obtained after centrifugation at 10,000×*g* for 5 min (4°C). The specific glucose consumption rate, defined as the differential change in glucose concentration with time, was calculated from the equation, *q*_glucose_ = µ/*Y_x_*, where µ is the specific growth rate (h^−1^) and *Y_x_* is the biomass yield (g.mol carbon substrate^−1^).

### Gene expression analysis by RT-PCR

RT-PCR was performed using SYBR Green technology on a Lightcycler instrument (Roche applied Science) as described previously [32]. The primers used in this study have been described previously [8].

### Purification of Rex

The *rex* ORF was amplified by PCR from *B. cereus* ATCC 14579 using the oligonucleotides, pET101*rex*F (5′-ACCATGGATCAGCAAAAGATTCCA-3′) and pET101*rex*R (5′-TTGTGGATAGTTTTTCAAAAAGTAAAC-3′). The amplicon was introduced as a blunt-end PCR product into pET101/D-TOPO (Invitrogen). The integrity of the inserted sequence was confirmed by DNA sequencing. The resulting construct was transformed into *E. coli* BL21-CodonPlus(DE3)-RIL strain (Stratagene) for protein production. BL21-CodonPlus(DE3)-RIL cells carrying the pET101-*rex* expression plasmid were grown in 1 L LB medium containing 100 µg.mL^−1^ ampicillin, at 30°C with agitation (200 rpm) until the cell density reached an OD_600_ of about 0.5. After this, 0.5 mM isopropyl-β-D-thiogalactopyranoside (IPTG) was added to the culture, and growth was continued for four hours. The cells were harvested by centrifugation (6500 rpm for 10 min at 4°C), washed, and resuspended in 20 mL ice-cold extraction buffer consisting of 50 mM TRIS buffered at pH 8.0, 0.3 M NaCl, 10% glycerol, and a protease inhibitor cocktail (one tablet of Complete Mini, EDTA free, Roche). Cells were then incubated with 0.5 mg/mL lysozyme for 45 min under gentle agitation at 4°C and disrupted by sonication for 5 min at 4°C using a Vibra Cell ultrasonicator (Fisher Bioblock Scientific). The lysates were clarified by centrifugation at 9000 rpm for 30 min at 4°C and loaded onto a 2 mL Co^2+^-immobilized TALON metal affinity chromatography column (Clontech) equilibrated with the extraction buffer. The column was washed with 30 mL of extraction buffer containing 20 mM imidazole and Rex was eluted with 3 mL extraction buffer containing 200 mM imidazole. The eluted fraction was desalted by dialysis, concentrated using Nanosep 30 kDa-molecular-mass cutoff devices (Omega disc membrane; Pall filtron), and stored at −80°C until analysis. The purity of the protein was estimated to be above 90% by Coomassie blue-stained SDS-PAGE. The protein concentration was determined using a Bradford assay (Interchim) with bovine serum albumin as the reference.

### Electrophoretic mobility shift assays (EMSAs)

Nucleic acid fragments containing the promoter regions of *ldhA* and *rex* were PCR-amplified using biotinylated forward primers, LdhAF (5′-ACCTGCTAATCCGATGATTG-3′) and RexF (5′-CAAGAATCGTTTCTGCACCG-3′), and nonbiotinylated reverse primers, LdhAR (5′-GGATCCAACTAATCCAGTAC-3′) and RexR (5′-GCTTACCAGAAAGAGATAAG-3′). The DNA used as negative control was a fragment of the ssuRNA BC0007 sequence (NC_004722), which was amplified with the biotinylated ssubioF (5′-GGTAGTCCACGCCGTAAACG-3′) and ssuR (5-GACAACCATGCACCACCTG-3′) primer pair. The 5′-labeled amplicons were purified using the High Pure PCR Product Purification Kit (Roche). Binding reactions were performed for 30 min at 37°C by incubating biotin-labeled DNA fragments (2 nM per reaction) with different amounts of Rex in 10 mM Tris-HCl buffered at pH 7.5, and containing 50 mM KCl, 2.5% glycerol, 5 mM MgCl_2_ and 5 mg/L poly(dI−dC). The samples were resolved by electrophoresis on a 6% nondenaturing polyacrylamide gel and electrotransferred onto Hybond N+ Nylon membranes (Amersham). Biotin-labeled DNA was detected using the LightShift Chemiluminescent EMSA Kit (Pierce).

### Proteomic sample preparation and nanoLC-MS/MS analysis of tryptic peptides

Three independent biological replicates were harvested for each of the two conditions (aerobiosis and anaerobiosis) and two strains (Δ*rex* mutant and its parent strain, ATCC 14579). The extracellular proteins of the 12 cultures were extracted by trichloroacetic acid precipitation [8]. The cellular proteins from the 12 samples were obtained as previously described [9]. The 24 resulting samples were subjected to SDS-PAGE, and then identified after trypsin proteolysis by nanoLC-MS/MS tandem mass spectrometry with an LTQ-Orbitrap XL mass spectrometer as previously described [8], [9]. A total of 131 nanoLC-MS/MS runs were carried out to acquire the whole dataset. The MS/MS spectra were assigned to tryptic peptide sequences with the Mascot Daemon software (version 2.3.2; Matrix Science) with mass tolerances of 5 ppm on the parent ion and 0.5 Da on the MS/MS, fixed modification for carbamidomethylated cysteine, and variable modification for methionine oxidation. Mascot results were parsed with a *p*-value threshold below 0.05 for peptide identification and proteins were validated when at least two peptides were detected. The number of MS/MS spectra per protein recorded by nanoLC-MS/MS was extracted for each sample. In each condition, proteins were further considered for comparison only if peptides were seen in at least two of the three replicates. The resulting datasets were normalized taking into account the total protein concentration of the corresponding pellet and supernatant. Protein concentrations in *B. cereus* were determined using the Reducing agent Compatible Detergent Compatible (RCDC) protein assay (Bio-Rad) following the supplier’s instructions. MS/MS spectral counts were compared with the TFold method using the PatternLab software program 2.0.0.13 [33] using a *p*-value cut-off set at 0.05. Log_2_ (fold-change) were then calculated for comparisons and only the proteins for which the *p*-value was lower than the 0.05 cut-off were considered. The mass spectrometry proteomics data have been deposited to the ProteomeXchange Consortium (http://www.proteomexchange.org) via the PRIDE partner repository [34] with the dataset identifier PXD000856 and DOI 10.6019/PXD000856.

### Exposure of bacteria to H_2_O_2_ and viability assays

Oxidative stress resistance of the *B. cereus* Δ*rex* mutant and the parent strain, ATCC 14579, was assessed by exposing aerobically grown (OD∼0.4) and anaerobically grown (OD∼0.2) cells to 20 and 5 mM H_2_O_2_, respectively. Samples were taken prior to oxidative stress (time zero) and after 20 min. Aliquots (100 µl) of the samples were diluted in H_2_O, appropriate dilutions of the culture were plated onto LB agar, and after overnight incubation at 37°C the colony forming units (CFUs) were counted. All the experiments were performed at least in triplicate, and at least two technical replicates from each dilution step were carried out to determine the number of CFUs.

## Results

### Expression analysis of *rex* in *B. cereus* ATCC 14579 at two different pO_2_

We identified BC_0291 in the *B. cereus* ATCC 14579 genome [27] as the homologue to the *Bacillus subtilis* transcriptional repressor, Rex [12], [35], [36], with 90% sequence identities. We established by 5′RACE PCR (Fig. S1) that a transcriptional start site (G) was located 41 bp from the translational start site (ATG) of the BC_0291 open reading frame. Upstream of this transcriptional start site, we identified a potential housekeeping σA-type promoter, TATACAN(17)TAAACT. The stop codon (TAA) overlapped an inverted repeat (AAAACGCAGAGG(N6)CCTCTGCGTTTT; ΔG = −23.2 kcal/mol) that may be a transcriptional terminator, suggesting that *rex* was monocistronically transcribed. Using real-time RT-PCR, we investigated the expression of *B. cereus rex* under aerobiosis and anaerobiosis in early-growing ATCC 14579 cells. The results indicated that *rex* expression was significantly higher under aerobiosis than under anaerobiosis (log_2_ fold-change = 1.1, *p*-value<0.05).

### Rex regulates glucose catabolism in *B. cereus*

**Figure 1:**
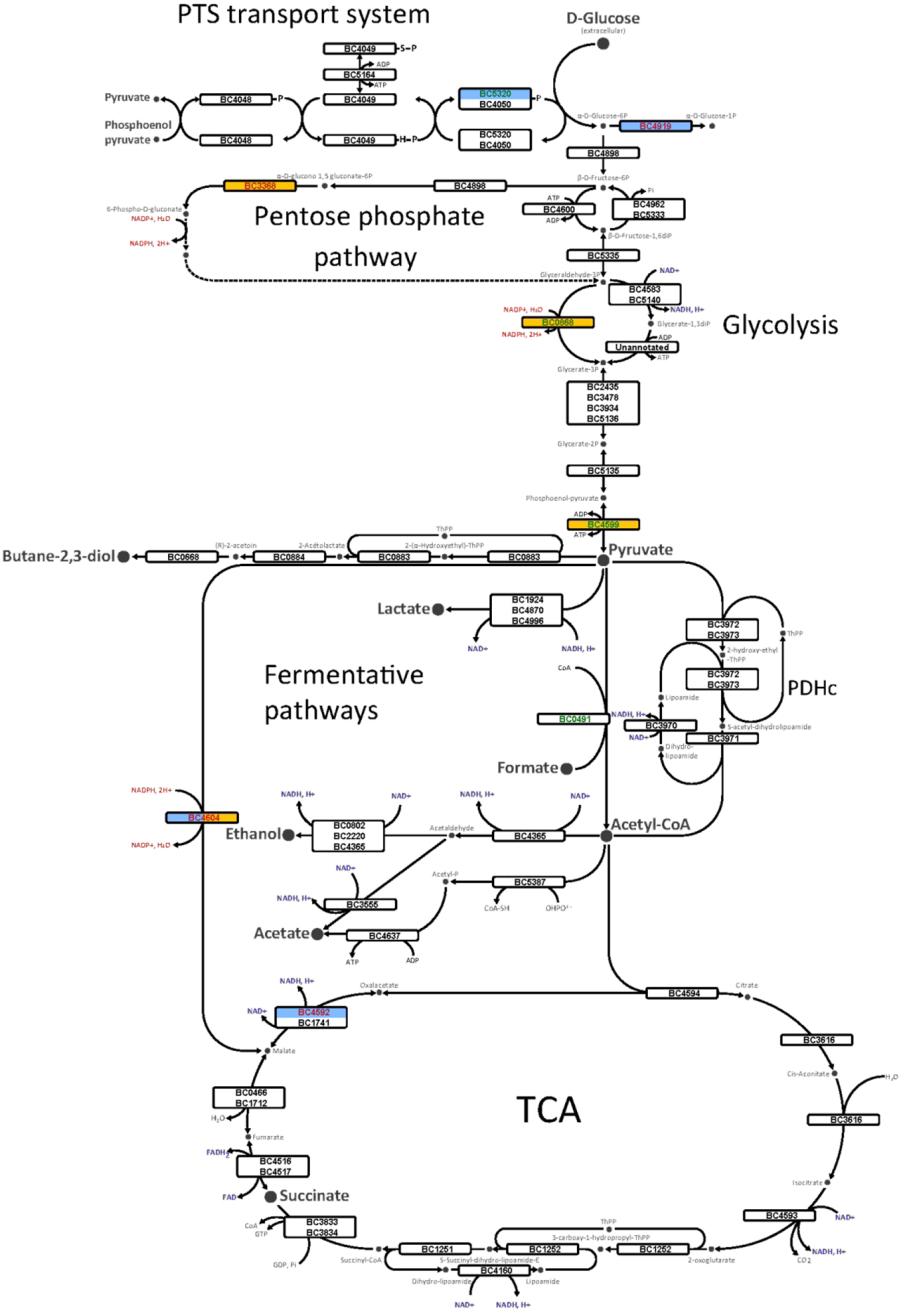
An overview of the main anaerobic and aerobic glucose catabolic pathways utilized by *B. cereus* ATCC 14579. The proteins detected in this study are indicated by their BC number. Protein names and functions are listed in Table S6. The form filling indicates fold-change values that satisfied the Student’s *t*-test statistical criteria (*p*-value<0.05) in anaerobiosis (blue) and aerobiosis (yellow). Red and green BC numbers indicated significant increase and decrease, respectively, of abundance level of the proteins in Δ*rex* mutant compared with wild-type. The nicotinamide nucleotides are indicated in red (NADP^+^/NADPH) or blue (NAD^+^/NADH).

Given that Rex orthologues function as NAD^+^/NADH-responsive regulators of metabolism in several Gram-positive bacteria (such as *B. subtilis*, *S. aureus* and *S. coelicolor* [12], [13], [14], [15], [36], [37]), we sought to determine if *B. cereus rex* contributed to anaerobic fermentative and aerobic respiratory growth. As shown in Table 1, *rex* deletion slightly increased the *B. cereus* growth rate under both anaerobiosis and aerobiosis without a significant change in final biomass. Under anaerobiosis, the increase of growth rate was not related to glucose consumption, suggesting perturbation of the fermentative pathways. In accordance with this hypothesis, the spectra of fermentation end products were significantly modified by the *rex* deletion (Table 1). We observed that formate, acetate and ethanol production were promoted at the expense of lactate production. In addition, the ethanol-to-acetate ratio increased in Δ*rex* mutant, while NADH recovery levels remained unchanged and ATP yields tended to increase. Under aerobiosis, respiratory Δ*rex* cells secreted ∼8-fold higher amounts of lactate compared with the wild-type cells, while producing lower levels of acetate. We conclude that *rex* deletion affects the carbon flux through the NADH recycling lactate pathway under both anaerobiosis and aerobiosis (Fig. 1). Complementation for growth of the non-polar *rex* mutant in *B. cereus* ATCC 14579 was not possible using a pHT304-based plasmid. This is probably due to the presence of multiple copies of the *rex* promoter region and/or overexpression of *rex* [38]. Therefore, to validate the role of Rex in aerobic respiration and anaerobic fermentation in *B. cereus*, we deleted the *rex* gene of *B cereus* F4430/73 [39] and cultured the mutant strain under both aerobiosis and anaerobiosis. The results showed that the growth phenotype of Δ*rex* was the same in strains ATCC 14579 and F4430/73 (Table S1).

**Table 1:** Results from controlled batch cultures of Δ*rex* mutant and its parent strain, *B. cereu*s ATCC 14579^a^.

	Anaerobic fermentative growth		Aerobic respiratory growth	
	WT^b^	Δ*rex*	WT^b^	Δ*rex*
Maximal specific growth rate (µ_max_) (h^−1^)	0.84±0.04	0.90±0.02*	1.22±0.11	1.41±0.04*
Final biomass (g.liter^−1^)	0.78±0.01	0.83±0.06	2.38±0.12	2.31±0.12
*Y_glucose_* (g of cells. mol of glucose^−1^)	26±1	28±1	79±1	77±1
Maximal specific glucose consumption(mmol.g^−1^.h^−1^)	32±2	32±2	15±1	18±2
Yields of end products (mol.mol glucose^−1^)^c^				
Lactate (*Y*_l*actate*_)	1.33±0.01	1.25±0.02*	0.05±0.02	0.40±0.01*
Acetate (*Y_acetate_*)	0.32±0.01	0.36±0.01*	1.05±0.01	0.69±0.03*
Formate (*Y_formate_*)	0.23±0.01	0.27±0.01*	0.01±0.01	0.01±0.01
Ethanol (*Y_ethanol_*)	0.13±0.01	0.18±0.01*	NZ^d^	NZ
Succinate (*Y_succinate_*)	0.01±0.01	0.01±0.01	0.05±0.01	0.04±0.01
Ethanol versus Acetate	0.40	0.50*		
ATP yield^e^	2.10	2.15	ND^f^	ND
NADH recovered^g^	1.2	1.2	ND	ND

a Cells were grown under anaerobiosis (pO_2_ = 0%) and full aerobiosis (pO_2_ = 100%). Data are the means of triplicate measures obtained from three independent cultures.

b WT, wild-type parent strain *B. cereus* ATCC 14579.

c Yields of end products were calculated at the stationary phase.

d NZ, yield was below 0.01 mol.mol glucose^−1^.

e ATP yield was calculated as moles of ATP produced per mole of consumed glucose, and was equal to *Y*_l*actate*_+*Y_ethanol_*+2*Y_acetate_.

f ND, not determined.

g NADH recovery was calculated as the ratio of pathways producing NADH versus those consuming NADH (producing NAD^+^), and was equal to (lactate+2×acetate+2×ethanol−formate)/(lactate+2×ethanol).

*p<0.05 *versus* WT in Student’s t-test.

### Rex regulates hydrogen peroxide resistance of *B. cereus* cells

**Figure 2:**
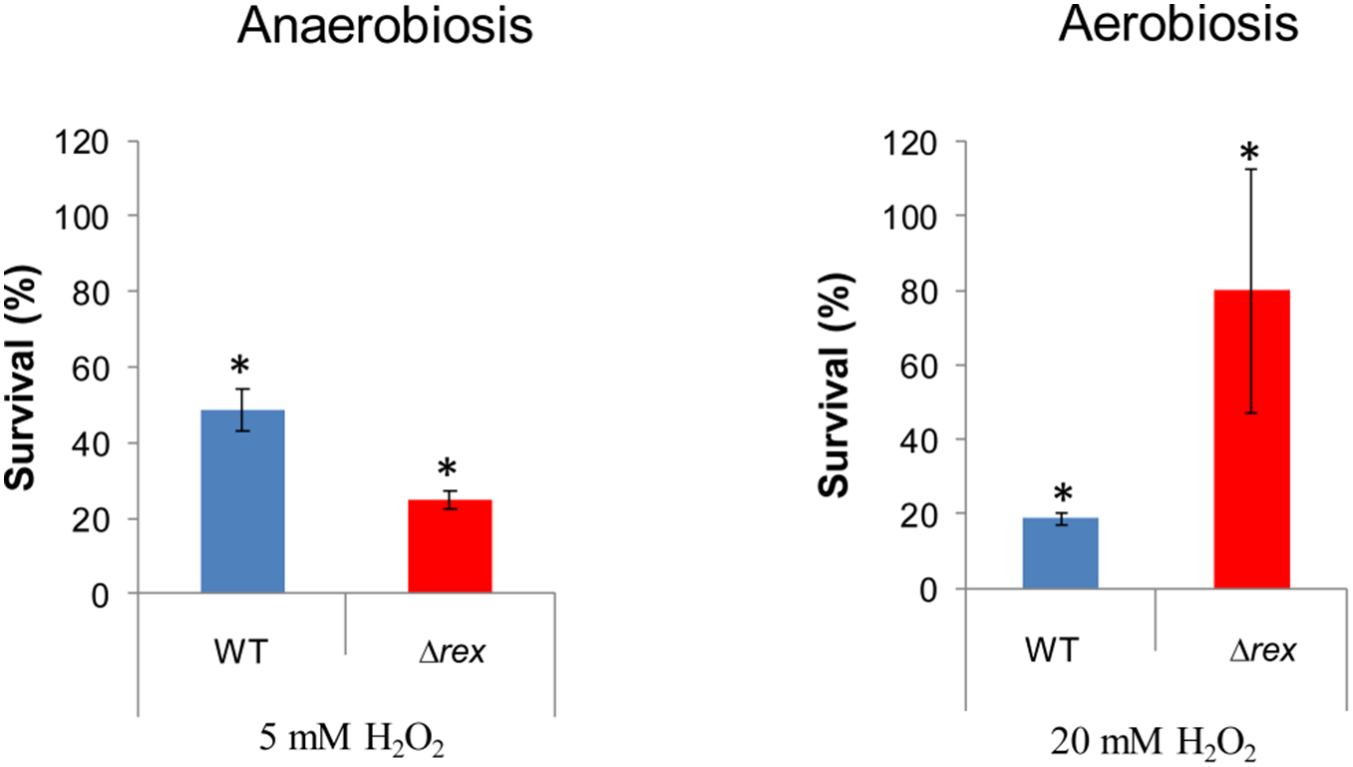
Survival of *B. cereus* cells towards external hydrogen peroxide under aerobiosis and anaerobiosis. Cells were grown in liquid cultures to the mid-exponential growth phase either aerobically or anaerobically, and subjected to 20 or 5 mM H_2_O_2_, respectively. Samples were taken at 0 and 20 min after H_2_O_2_ addition. Colony forming units per mL were counted and expressed as (N/No)×100. Error bars represented the standard deviation from three independent measures. Significant differences (*p*-value<0.05) between mutant and wild-type strains are indicated with asterisks.

To evaluate the impact of Rex deficiency on *B. cereus* resistance to oxidative stress, aerobically and anaerobically grown ATCC 14579 cells were exposed to hydrogen peroxide (20 and 5 mM H_2_O_2_, respectively). Figure 2 shows that aerobically grown Δ*rex* cells were less susceptible to H_2_O_2_ harmful effects than WT cells, while the anaerobically grown Δ*rex* cells were more susceptible. These data indicate that Rex restricts the resistance to oxidative stress of aerobically grown *B. cereus* cells, while sustaining a high resistance to oxidative stress of anaerobically growing cells. To validate the role of Rex in the *B. cereus* resistance to external H_2_O_2,_ we repeated the experiments using the F4430/73 Δ*rex* strain (Fig. S2). The results showed that Rex deficiency similarly impacts F4430/73 and ATCC 14579 cells while the survival of F4430/73 anaerobically grown cells was strongly higher than the survival of ATCC 14579 cells.

### DNA binding activity of *B. cereus* Rex

**Figure 3:**
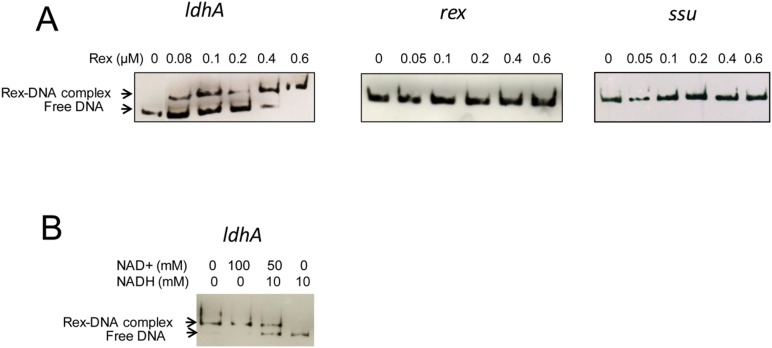
*In vitro* binding of Rex to *ldhA* promoter region determined by EMSA. **Panel A**. Biotin-labeled PCR products (2 nM) corresponding to *ldhA* and *rex* promoter regions and a negative control (a fragment of ssuRNA BC_0007 sequence) were mixed with increasing concentrations of purified Rex. **Panel B**. PCR product corresponding to the *ldhA* promoter region was incubated with 0.6 µM purified Rex and different concentrations of NAD^+^ and NADH, as indicated.

Previous studies have shown that proteins belonging to the Rex family function as dimers that bind to promoter regions containing DNA motifs with the 5′-TTGTGAAnnnnTTCACAA-3′ consensus sequence [21]. This typical binding motif is missing in the *rex* regulatory region of *B. cereus*, as reported for *S. aureus* and *B. subtilis* [12], [15]. However, a putative binding motif with two mismatches compared with the known consensus motif was found upstream of the transcription start site of *ldhA* [25] in *B. cereus,* as in *S. aureus* and *B. subtilis*. To test whether *B. cereus* Rex binds to the *ldhA* promoter region, we overexpressed *B. cereus rex* in *E. coli*, purified the dimeric His6-tagged recombinant protein (Fig. S3), and performed electrophoretic mobility shift assays (EMSA). As a negative control, we assessed the binding of the Rex protein to the *ssu* DNA fragment (Fig. 3A). The results showed that a concentration of 0.6 µM Rex led to a complete shift of the *ldhA* fragment (2 nM). As expected, no shift was observed with the *rex* DNA fragment. The specificity of the binding was demonstrated by the absence of DNA shift with the negative control. The second EMSA experiment (Fig. 3B) showed that 10 mM NADH impaired Rex binding to the *ldhA* promoter region while 100 mM NAD^+^ did not interfere with Rex-DNA complex formation. An NAD^+^/NADH ratio of five led to an incomplete shift of the *ldhA* fragment. We conclude that in *B. cereus*, as in *B. subtilis* and *S. aureus* [15], Rex binding activity depends on the NADH/NAD^+^ ratio.

### Global comparative proteomics of *B. cereus* Δ*rex* and wild-type cells

To investigate the regulatory role of Rex in aerobic respiratory and anaerobic fermentative growth of *B. cereus* and decipher its role in toxinogenesis, we carried out a comparative proteome analysis from cells harvested in exponential growth phase (µ = µ_max_). We compared the cellular and extracellular proteomes of aerobically and anaerobically grown Δ*rex* with those of its parental strain, with biological triplicates for each condition. A total of 1,620 proteins were identified in the whole study (Tables S2, S3, S4 and S5), with 1,450 proteins specifically detected in the whole-cell shotgun analysis and 170 further proteins observed in the exoproteome study. These proteins were quantified by spectral count as previously described [8], [9]. The Rex-dependent changes in protein abundance, expressed as log_2_ of the ratio of a protein’s abundance at a given condition (aerobiosis or anaerobiosis) relative to the wild-type, are presented in Tables S6 and S7, where the proteins with significant abundance changes based on the PatternLab Tfold student t-test (*p*-value below 0.05) are highlighted while proteins with non-significant changes are in grey color.

#### (i) Anaerobiosis

Among the whole cellular proteome (Table S6), 145 proteins showed significant abundance level changes in Δ*rex* cells relative to WT (*p*-values<0.05): 89 proteins were significantly up-regulated and 56 proteins were significantly down-regulated in Δ*rex* cells. In the exoproteome (Table S7), 41 proteins showed significant abundance level changes in Δ*rex* cells relative to WT: 17 proteins were significantly up-regulated and 24 proteins were significantly down-regulated in Δ*rex* cells.

#### (ii) Aerobiosis

Table S6 indicates that 132 proteins showed significant abundance level changes in Δ*rex* cellular proteome (*p*-values<0.05): 82 proteins were significantly up-regulated and 50 proteins were significantly down-regulated in Δ*rex* cells. Table S7 indicates that 70 proteins showed significant abundance level changes in Δ*rex* cellular exoproteome (*p*-values<0.05): 35 were significantly up-regulated and 35 were significantly down-regulated in Δ*rex* cells.

#### (iii) Global analysis (Fig S4)

Only 8 cellular proteins showed similar behavior under both aerobiosis and anaerobiosis (4 were up-regulated and 4 were down-regulated in Δ*rex* cells). Only 3 extracellular proteins were down-regulated under both anaerobiosis and aerobiosis. However, all the extracellular proteins that were up-regulated under anaerobiosis were also up-regulated under aerobiosis. Taken together, the data indicate that Rex (i) differently modulates the cellular proteome of *B. cereus* anaerobically and aerobically grown cells in terms of the identity of proteins that show significant abundance level changes, (ii) acts mainly as a repressor at the cellular proteome level under both aerobiosis and anaerobiosis and, has a stronger impact on the exoproteome of aerobically grown than anaerobically grown cells.

### Insights into the cellular proteome of the Δ*rex* mutant

The list of cellular proteins showing significant abundance changes (*p*-values<0.05) due to the absence of Rex includes proteins related to glucose metabolism, amino acid, nucleotide and lipid metabolism, protein folding, response to stress, virulence, cell wall/membrane assembly, transport, and a number of proteins of unknown function under both aerobiosis and anaerobiosis (see Table S6 for details on protein characteristics, the fold-changes and *p*-values in each comparison).

#### (i) Impact of Rex on glucose metabolism-related proteins under anaerobic fermentative conditions

Figure 1 shows an overview of the glucose catabolic pathways of *B. cereus*. As illustrated in Fig. 1, the abundance level of key fermentative enzymes, such L-lactate dehydrogenases (Ldh; BC_1924, BC_4870, BC_4996), pyruvate formate lyase (PflB; BC_0491), alcohol dehydrogenase (BC_4365, BC_2220, BC_0802) and acetate kinase (BC_4634) were not significantly different in the mutant compared with wild-type cells. However, the lack of Rex resulted in a decrease of the abundance of the IIA component of the glucose-specific phosphotransferase (PTS) system (BC_5320, log_2_ = −0.4; *p*-value = 0.021). A significant decrease in abundance was also observed for the pyruvate-formate-lyase-activating enzyme, PflA (BC_0492; log_2_ = −1; *p*-value = 0.048). PflA is the sole enzyme able to activate the oxygen-sensitive PflB (BC_0491), which converts pyruvate to acetyl-CoA and formate (Fig. 1) [40]. In contrast, anaerobically grown Δ*rex* cells sustain a higher level of phosphoglucomutase (Pgm; BC_4919) than the wild-type cells (log_2_ = 0.6; *p*-value = 0.036) and thus, could sustain a higher carbon flux through gluconeogenesis than glycolysis (Fig. 1). As for Pgm, the malate dehydrogenase (Mdh; BC_4592) abundance level was increased in the absence of Rex under anaerobiosis (log_2_ = 1.7; *p*-value = 0.017). Mdh is one of the enzymes involved in the NADH-dependent reduction of oxaloacetate to malate, which is the first reaction in the succinate pathway (Fig. 1). The increase of Mdh abundance in the Δ*rex* mutant compared with wild-type was associated with the increase of NADPH-producing malic enzyme (ME; BC_4604; log_2_ = 0.5; *p*-value = 0.033), which converts malate into pyruvate. Taken together, our data suggest that Rex may modulate the abundance level of key metabolic enzymes to control carbon flow through fermentative pathways, possibly at the expense of the pathway that couples NADH-recycling Mdh with NADPH-producing ME.

#### (ii) Impact of Rex on glucose metabolism-related proteins under aerobic respiratory conditions

Figure 1 shows that ME was the sole enzyme showing an abundance level change in both aerobically and anaerobically grown mutant cells (log_2_ = 0.4; *p*-value = 0.01 under aerobiosis). 6-Phosphogluconolactonase (Pgl; BC_3368) showed a higher increase than ME (log_2_ = 1.3; *p*-value = 0.047) in Δ*rex* aerobically grown cells. The increase of the Pgl abundance level may have an impact on the activity of the NADPH-generating pentose phosphate pathway (PPP) in the Δ*rex* mutant (Fig. 1). Two glycolytic proteins showed significant abundance decreases in aerobically grown mutant cells: the NADPH-producing glyceraldehyde-3-phosphate-dehydrogenase (NADPH-GAPDH; BC_0868; (log_2_ = −0.5; *p*-value = 0.012) and pyruvate kinase (PK; BC_4599; (log_2_ = −0.4; *p*-value = 0.032), which catalyzes the last steps of glycolysis. The decrease of the NADPH-GAPDH level could increase the carbon flow through the NADH-producing GAPDH (BC_4583, BC_5333) and thus stimulate flux through the upstream glycolysis pathways, especially through the NADH-recycling lactate pathway (Fig. 1). Finally, Rex may modulate glycolytic flux by controlling the abundance level of key enzymes of glycolysis and pentose phosphate pathway when cells are grown under aerobiosis in glucose-containing medium.

#### (iii) Impact of Rex on other relevant cellular pathways

*Cell-wall and cell surface related-proteins.* Among the 20 proteins classified in the cell-wall/surface functional group, 8 showed significant abundance level changes (p<0.05) in anaerobically grown Δ*rex* mutant cells while 4 proteins showed significant abundance level changes in aerobically grown cells (Table 2). A protein annotated as a putative prolipoprotein diacylglyceryl transferase (Lgt; BC_5163), which may be required for lipid modification of the cysteine residue present within the lipobox of prolipoproteins [41], showed a significant change of abundance level under both conditions: it was more highly detected in anaerobically grown cells and less detected in aerobically grown cells when Rex was absent.

**Table 2:** Cellular abundance level changes of cell membrane, transport, stress response, transcriptional regulation, virulence and phage-related proteins in Δ*rex* mutant relative to wild-type under anaerobiosis and aerobiosis.

				Abundance level change and significance			
				Anaerobiosis		Aerobiosis	
Accession no (NP)	Proteinname	Gene number	Functionnal annotation	log_2_(fold-change)^a^	*p*-value	log_2_(fold-change)	*p*-value
			**Cell wall, membrane and cell surface proteins**				
NP_833630.1	-	BC_3910	N-acetylglucosaminyl transferase	**1,561**	**0,018**	−1,515	0,197
NP_835080.1	TagA	BC_5419	N-acetyl-beta-D-mannosaminyltransferase	**1,263**	**0,042**	−0,377	0,382
NP_832677.1	-	BC_2929	Peptidoglycan N-acetylglucosamine deacetylase	**0,832**	**0,019**	0,799	0,183
NP_835088.1	-	BC_5427	Cell wall biosynthesis glycosyltransferase	−**1,120**	**0,046**	−0,889	0,070
NP_834932.1	AmsK	BC_5269	Amylovoran biosynthesis AmsK	−0,713	0,112	**1,595**	**0,001**
NP_834404.1	-	BC_4698	Choline kinase	**0,949**	**0,032**	0,214	0,199
NP_834827.1	Lgt	BC_5163	Prolipoprotein diacylglyceryl transferase	**1,007**	**0,012**	−**0,811**	**0,004**
NP_834936.1	-	BC_5273	UDP-bacillosamine synthetase	**1,322**	**0,033**	0,632	0,110
NP_835093.1	-	BC_5432	Bactoprenol glucosyl transferase	−**1,218**	**0,032**	0,536	0,306
NP_835019.1	-	BC_5358	Collagen adhesion protein	0,918	0,088	**1,170**	**0,045**
NP_830682.1	-	BC_0896	S-layer protein	−0,234	0,180	−**0,286**	**0,017**
			**Transport-related proteins**				
NP_829996.1	SecE	BC_0115	Preprotein translocase subunit	−**0,971**	**0,044**	0,411	0,252
NP_834447.1	-	BC_4743	ABC transporter ATP-binding protein	**1,561**	**0,018**	−0,029	0,485
NP_834539.1	-	BC_4839	ABC transporter ATP-binding protein	**1,585**	**0,031**	0,856	0,115
NP_830141.1	Uup	BC_0290	ABC transporter ATP-binding protein	0,151	0,382	**0,705**	**0,045**
NP_830187.1	-	BC_0348	Methionine ABC transporter	−**1,690**	**0,004**	2,160	0,064
NP_830967.1	OppD	BC_1182	Oligopeptide transport ATP-binding protein	**0,651**	**0,002**	−0,044	0,421
NP_830660.1	-	BC_0874	Arginine ABC transporter ATP-binding protein	−0,396	0,349	−**1,218**	**0,024**
NP_832817.1	CutC	BC_3071	Copper tansport	**0,903**	**0,007**	−0,358	0,341
NP_830432.1	-	BC_0615	Di-/tripeptide transporter	−**0,837**	**0,047**	0,422	0,232
NP_830429.1	-	BC_0612	L-lactate permease	**0,748**	**0,026**	−0,644	0,261
			**Stress response-related proteins**				
NP_831381.1	**-**	BC_1603	Cold shock protein	−**1,120**	**0,037**	−0,474	0,277
NP_830286.1	TelA	BC_0447	Tellurite resistance protein	0,084	0,432	**0,840**	**0,008**
NP_833675.1	-	BC_3956	GTP-binding protein TypA/BipA	**0,310**	**0,049**	0,043	0,419
NP_833972.1	-	BC_4258	Hydroxyacylglutathione hydrolase	**0,824**	**0,028**	−0,578	0,213
NP_833576.1	-	BC_3855	Alkaline-shock protein	0,014	0,488	**0,740**	**0,026**
NP_834187.1	OhrA	BC_4475	Organic hydroxyperoxide protein A	0,189	0,340	**2,029**	**0,015**
NP_829959.1	Hsp15	BC_0062	Heat shock protein 15	0,799	0,082	**1,667**	**0,007**
NP_830216.1	AhpC	BC_0377	Alkyl hydroperoxide reductase C22	−0,136	0,426	−**1,029**	**0,007**
NP_830941.1	KatE	BC_1155	Catalase	**1,111**	**0,014**	−0,074	0,466
NP_834345.1	-	BC_4639	Thiol peroxidase	−0,044	0,463	−**0,737**	**0,034**
			**Transcriptional Regulation**				
NP_833888.1	ArgR2	BC_4174	Arginine biosynthesis repressor	**1,511**	**0,030**	1,269	0,079
NP_833771.1	-	BC_4053	Transcriptional regulator, GntR family	**0,895**	**0,019**	0,227	0,399
NP_833799.1	-	BC_4081	MarR family transcriptional regulator	0,029	0,464	**1,903**	**0,001**
NP_834861.1	-	BC_5197	MarR family transcriptional regulator	**0,345**	**0,029**	0,604	0,123
NP_832113.1	-	BC_2351	MerR family transcriptional regulator	0,485	0,276	**1,170**	**0,045**
NP_830532.1	TenA	BC_0742	Transcriptional activator	**0,791**	**0,046**	−0,621	0,193
NP_834137.1	-	BC_4425	Transcriptional regulator	**0,632**	**0,044**	−1,029	0,051
NP_831310.1	-	BC_1531	Transcriptional regulatory protein	−0,029	0,471	**1,480**	**0,004**
NP_831256.1	ResD	BC_1477	Transcriptional regulatory protein	**0,356**	**0,008**	−0,599	0,119
NP_830596.1	-	BC_0806	BigG family transcription antiterminator	0,485	0,276	**1,922**	**0,000**
			**Virulence-related proteins**				
NP_834769.1	HlyI	BC_5101	Hemolysin I	−0,152	0,438	**2,611**	**0,001**
NP_832488.1	Npr2	BC_2735	Bacillolysin	−1,089	0,193	−**2,120**	**0,013**
NP_833332.1	HhoA	BC_3600	Protease	0,485	0,098	−**1,690**	**0,026**
NP_831147.1	-	BC_1366	SSEB protein	**1,163**	**0,036**	1,269	0,055
			**Phage-related proteins**				
NP_831667.1	-	BC_1894	Phage protein	**3,023**	**0,014**	−1,358	0,286
NP_831632.1	-	BC_1859	Phage protein	**2,526**	**0,039**	−0,644	0,261
NP_831635.1	-	BC_1862	Phage protein	**1,714**	**0,027**	−0,761	0,302
NP_831673.1	-	BC_1901	Phage protein	**1,727**	**0,012**	−1,889	0,205
NP_831676.1	-	BC_1904	Phage protein	**1,975**	**0,038**	−0,515	0,367

Only changes satisfying statistical criteria (*p*-value<0.05) at least in one growth condition are shown.

a The relative amount of each protein was determined using PatternLab software. Numbers in bold indicate that satisfied statistical criteria. See Table S6 for details on all the proteins showing significant abundance level changes in Δ*rex* cells.

*Transport-related proteins*. The largest change in transport-related protein abundance was observed in anaerobically grown Δ*rex* cells: 8 proteins showed significant abundance level changes (p<0.05). This suggests that the Rex-dependent control of the abundance pattern of transport related-proteins was stronger in anaerobic fermentative conditions than in aerobic respiratory conditions.

*Transcriptional regulation-related proteins.* Δ*rex* up-regulated the synthesis of 6 and 4 transcriptional regulators under anaerobiosis and aerobiosis, respectively. Among these, we found regulators of the Mer and Mar families, which control many cellular processes including oxidative stress response and virulence [43], [44]. Interestingly, we noted that ResD was significantly up-regulated in anaerobically grown Δ*rex* cells but remained unchanged in aerobically grown Δ*rex* cells. ResD is involved in both metabolism and toxinogenesis in *B. cereus* [22], [32].

*Stress-response related proteins*. Among the stress response-related proteins, the antioxidant protein, OhrA [7], [9], was significantly up-regulated in aerobically grown Δ*rex* cells and its abundance remained constant under anaerobiosis (Table 2). Like OhrA, the ribosome-associated heat-shock protein, Hsp15 (BC_0062), showed, under aerobiosis, a significant change in abundance level. This protein could be involved in the recycling of free 50 S ribosomal subunits [45]. Interestingly, we noted significant changes in the abundance patterns of 50 S ribosomal subunits in aerobically grown Δ*rex* cells (Table S6). Unlike aerobically grown cells, anaerobically grown Δ*rex* cells exhibited higher levels of catalase, KatE (BC_1155), lower levels of the BC1603 protein, which is functionally annotated as a cold-shock protein and no significant level change of alkyl hydroperoxide reductase (AhpC, BC_0377). Clearly, Rex controls the abundance pattern of stress-related proteins in an oxygen-dependent manner.

*Phage-related proteins.* Five phage-related proteins were strongly up-regulated in anaerobically grown Δ*rex* cells (log_2_>1.5, *p*-value<0.05) and their abundance remained constant in aerobically grown Δ*rex* cells. The functions of these proteins are unknown but they are considered to be potentially beneficial to *B. cereus* in facing adverse environmental conditions [42].

*Virulence-related proteins.* Table 2 shows that *rex* deletion has a significant impact on the abundance levels of hemolysin I (Hly I), neutral protease Npr2 (BC_2735), protease HhoA (BC_3600), and SSEB (BC_1366) proteins under aerobiosis, all of which are predicted to be secreted virulence factors [4], [46].

### Insights into the extracellular proteome of the Δ*rex* mutant

#### (i) Focus on toxin-related proteins

Although the intracellular abundance level of HlyI was higher in the aerobically grown Δ*rex* mutant strain, the extracellular level of this protein was not significantly different in the mutant compared with wild-type cells (Table S7). This was also the case for EntB, a putative enterotoxin [9]. However, 12 other toxin-related proteins showed a significantly higher abundance level in the exoproteome of aerobically grown Δ*rex* cells than wild-type cells (Table 3). Some of these proteins also showed a higher abundance level under anaerobiosis, but to a lesser extent, *i.e.* the three components of Nhe (NheA, NheB and NheC), EntA, EntC and the L1 component of Hbl. From these data, we conclude that Rex regulates the toxinogenic profile of the *B. cereus* exoproteome in response to oxygen availability.

**Table 3:** Changes in mRNA and protein abundance of toxins and putative toxins induced by Δ*rex* mutation under anaerobic and aerobic growth conditions^#^.

Protein name		Log_2_(fold-change) in *B. cereus* Δ*rex* vs wild-type^a^					
	Gene	Anaerobiosis			Aerobiosis		
		mRNA	Protein		mRNA	Protein	
			cellular	extracellular	s	cellular	extracellular
CytK	BC_1110	NS	NS^a^	NS	NS	NS	**1,72 (** ***p*** ** = 0.001)**
EntA	BC_5239	NS	NS	**0,29 (** ***p*** ** = 0.035)**	NS	NS	**0,53 (** ***p*** ** = 0.001)**
EntB	BC_2952	NS	NS	NS	NS	NS	NS
EntC	BC_0813	NS	NS	**0,37 (** ***p*** ** = 0.042)**	−**1,29 (p<0.05)**	NS	**0,75 (** ***p*** ** = 0.002)**
EntFM	BC_1953	NS	NS	NS	−**1,49 (p<0.05)**	NS	**0,68 (** ***p*** ** = 0.000)**
HblB	BC_3102	NS	NS	NS	NS	NS	**1,38 (** ***p*** ** = 0.001)**
HblB'	BC_3101	NS	NS	NS	NS	NS	**1,28 (** ***p*** ** = 0.002)**
HblL1	BC_3103	NS	NS	**0,32 (** ***p*** ** = 0.039)**	NS	NS	**1,09 (** ***p*** ** = 0.006)**
HblL2	BC_3104	NS	NS	NS	NS	NS	**1,29 (** ***p*** ** = 0.000)**
HlyI	BC_5101	NS	NS	NS	NS	**2,61(** ***p*** ** = 0.001)**	NS
HlyII	BC_3523	NS	NS	NS	NS	NS	**2,45 (** ***p*** ** = 0.000)**
NheA	BC_1809	NS	NS	**0,47 (** ***p*** ** = 0.003)**	NS	NS	**1,35 (** ***p*** ** = 0.002)**
NheB	BC_1810	NS	NS	**0,42 (** ***p*** ** = 0.008)**	NS	NS	**1,34 (** ***p*** ** = 0.001)**
NheC	BC_1811	NS	NS	**0,83 (** ***p*** ** = 0.001)**	NS	NS	**1,70 (** ***p*** ** = 0.001)**

a Concerning mRNA levels, each log_2_(fold-change) represents the mean level of mRNA in the Δ*rex* mutant samples relative to the mean level in the wild-type sample. The mean values were obtained from three measurements done on triplicate independent cultures. Only significant log_2_ ratios are indicated in bold. Concerning protein levels, the relative amount of each protein in Δ*rex* mutant compared to its parental strain was determined using PatternLab software. Numbers in bold indicate data satisfied statistical criteria (*p*-value<0.05). The *p*-values were indicated in brackets. Plus and minus indicate increased and decreased abundance levels, respectively. NS: no significant change was observed. For details, see Tables S6 and S7.

#### (ii) Other predicted extracellular proteins

Like the toxin-related proteins, many degradative enzymes, flagella components and proteins that are released from the cell wall, are more abundant in the exoproteome of the Δ*rex* mutant strain than wild-type cells, especially under aerobiosis (Table 4). All of these proteins are predicted to be extracellular proteins and, except for flagella components, all of them encompass signal peptides in their N-terminal primary structure and/or transmembrane helices. One metabolism-related protein, the subunit α of F0F1 ATP synthase (AtpA), and three proteins of unknown function (BC_1894, BC_5360 and BC_4062) also showed a higher abundance level in the exoproteome of aerobically grown Δ*rex* cells. Except for BC_1894, which was annotated as a prophage protein and thus could be secreted via holin(-like) pathway [47], these proteins contain a signal peptide, and thus are predicted to be secreted by classical export pathways.

**Table 4:** Changes in protein abundances in Δ*rex* exoproteome compared to wild-type exoproteome under anaerobiosis and aerobiosis.

							Log_2_ fold-change and significance^a^			
Proteinname	Gene	Accession no. (NP)		Functional annotation	TM domain	Export Signal peptide	Anaerobiosis		Aerobiosis	
							log_2_-fold-change	*p*-value	log_2_-fold-change	*p*-value
Degradative enzymes and adhesins										
ColC	BC_0556		NP_830373	Collagenase	N	Y	0,4	0,03	1,9	0,00
NprB	BC_5351		NP_835012	Bacillolysin	N	Y	0,67	0,00	1,97	0,00
NprP2	BC_2735		NP_832488	Bacillolysin	N	Y	1,58	0,00	2,6	0,01
HhoA	BC_3600		NP_833332	Protease	N	Y	0,176	0,241	0,6	0,03
Tgc	BC_1991		NP_831760	Putative murein endopeptidase	N	Y	0,45	0,01	1,71	0,00
PlcB	BC_0670		NP_830483	phospholipase C	N	Y	0,6	0,00	2,25	0,00
PlcA	BC_3761		NP_833485	Phosphatidylinositol phosphodiesterase	N	Y	0,48	0,02	2,55	0,00
InhA2	BC_0666		NP_830479	Immune inhibitor A precursor	N	Y	1,59	0,01	3,43	0,00
PgdA	BC_2929		NP_832677	Peptidoglycan deacetylase	N	Y	0,585	0,055	0,87	0,04
Sph	BC_0671		NP_830484	Sphingomyelin phosphodiesterase	N	Y	0,82	0,03	2,82	0,00
Flagella components										
FlaA	BC_1659		NP_831436	Flagellin	N	N	0,516	0,117	0,6	0,02
FlgK	BC_1636		NP_831414	Flagellar hook-associated protein	N	N	0,96	0,01	1,04	0,05
FlgL	BC_1637		NP_831415	Flagellar hook-associated protein	N	N	0,78	0,04	1,28	0,00
FliD	BC_1638		NP_831416	Flagellar capping protein	N	N	0,526	0,063	1,22	0,00
FlgB	BC_1641		NP_831419	Flagellar basal body rod protein	N	N	0,111	0,411	0,9	0,01
Cell wall and membrane-related proteins										
FtsI	BC_4270		NP_833984	Penicillin-binding protein	Y	N	0,189	0,145	1,73	0,02
YvgJ1	BC_2932		NP_832680	Phosphoglycerol transferase	Y	N	−0,03	0,462	1,25	0,03
YvgJ2	BC_5232		NP_834895	phosphoglycerol transferase	Y	N	−0,03	0,463	0,71	0,00
CwlD	BC_5196		NP_834860	N-acetylmuramoyl-L-alanine amidase	N	Y	0,485	0,022	0,67	0,00
CwlB	BC_0902		NP_830688	N-acetylmuramoyl-L-alanine amidase	N	Y	1,091	0,187	−1,1	0,04
Metabolism										
Eno	BC_5135		NP_834803	Phosphopyruvate hydratase	N	N	−1,2	0,01	−0,12	0,385
PfkA	BC_4600		NP_834306	6-Phosphofructokinase	N	N	−0,9	0,04	−0,18	0,377
PykA	BC_4599		NP_834305	Pyruvate kinase	N	N	−0,15	0,429	−4,6	0,01
Pgi	BC_4898		NP_834571	Glucose-6-phosphate isomerase	N	N	−1	0,04	0,014	0,493
AtpA	BC_3230		NP_832971	F0F1 ATP synthase subunit alpha	N	Y	−0,03	0,448	1,75	0,00
GapA	BC_5140		NP_834805	Glyceraldehyde-3-phosphate dehydrogenase	N	N	−0,62	0,144	−2,9	0,00
PtA	BC_5387		NP_835048	Phosphotransacetylase	N	N	−1,6	0,01	−2,1	0,03
GapN	BC_0868		NP_830654	NADP-dependent glyceraldehyde-3-phosphate dehydrogenase	N	N	−3,6	0,00	−1,32	0,054
FbA	BC_5335		NP_834997	Fructose-bisphosphate aldolase	N	N	−0,76	0,298	−3,6	0,03
Hpr	BC_4049		NP_833767	Phosphocarrier protein HPr	N	N	−2,4	0,03	−1,18	0,079
Bdh	BC_0868		NP_830481	(R,R)-butanediol dehydrogenase	N	N	−3,32	0,06	−3,6	0,02
PtsA	BC_4048		NP_833766	Phosphotransferase	N	N	−2,12	0,058	−3,2	0,00
SfcA	BC_4604		NP_834310	NAD-dependent malic enzyme	N	N	−0,86	0,187	−1,6	0,04
GlmM	BC_0188		NP_830056	Phosphoglucosamine mutase	N	N	−1,32	0,058	−3,8	0,00
GdhA	BC_4162		NP_833877	Leucine dehydrogenase	N	N	−1,8	0,04	−2,4	0,081
-	BC_4366		NP_834078	Cystathionine beta-lyase	N	N	0,151	0,447	−2	0,03
AdsS	BC_5468		NP_835123	Adenylosuccinate synthetase	N	N	−1,8	0,04	−1,15	0,202
-	BC_3799		NP_833521	Aspartate-semialdehyde dehydrogenase	N	N	−2,56	0,079	−3,2	0,01
ThiG	BC_0749		NP_830539	Thiazole synthase	N	N	ND	ND	−2,2	0,01
-	BC_0071		NP_829966	Phosphoribosyltransferase	N	N	−0,23	0,425	−3,5	0,00
DeoD	BC_1463		NP_831242	Purine nucleoside phosphorylase	N	N	−2,6	0,02	−1,06	0,185
-	BC_0015		NP_829919	Pyridoxine biosynthesis protein	N	N	−1,8	0,01	−3,6	0,00
-	BC_3981		NP_833700	tetrahydrodipicolinate -acetyltransferase	N	N	ND	ND	−3,3	0,00
MoaB	BC_4758		NP_834462	Molybdenum cofactor	N	N	−0,94	0,187	−2,3	0,02
FrvB	BC_4571		NP_834277	Deblocking aminopeptidase	N	N	−3,2	0,00	−0,92	0,215
PepT	BC_4143		NP_833858	Peptidase T	N	N	−1,6	0,04	−1,94	0,061
Stress-related proteins										
AhpC	BC_0377		NP_830216	Alkyl hydroperoxide reductase C22	N	N	−0,94	0,154	−2,2	0,00
GrpE	BC_4313		NP_834025	Chaperon	N	N	−1,4	0,04	−0,38	0,335
GroEL	BC_0295		NP_830146	Chaperon	N	N	−0,69	0,214	−2,3	0,01
Dps2	BC_5044		NP_834714	Non-specific DNA-binding protein	N	N	−2,4	0,00	−1	0,165
Dps1	BC_2011		NP_831779	Non-specific DNA-binding protein	N	N	−1	0,04	0	0,499
Sod	BC_5445		NP_835106	Superoxide dismutase [Mn]	N	N	−2,4	0,03	−0,92	0,226
AhpF	BC_0376		NP_830215	Alkyl hydroperoxide reductase subunit F	N	N	ND	ND	−2,4	0,02
TerD	BC_0443		NP_830282	tellurium resistance protein	N	N	−2,7	0,00	−0,97	0,064
Translation										
RpsF	BC_5476		NP_835129	30 S ribosomal protein S6	N	N	−2,3	0,01	−2,32	0,073
RpsG	BC_0126		NP_830007	30 S ribosomal protein S7	N	N	0,566	0,2	−1,8	0,01
RpsH	BC_0145		NP_830025	30 S ribosomal protein S8	N	N	1,709	0,059	−1,8	0,00
RpsJ	BC_0130		NP_830010	30 S ribosomal protein S10	N	N	ND	ND	−2,7	0,01
RplJ	BC_0119		NP_830000	50 S ribosomal protein L10	N	N	−0,58	0,16	−4,3	0,00
RplK	BC_5075		NP_834743	50 S ribosomal protein L11	N	N	−0,94	0,187	−2,4	0,02
RplL	BC_0120		NP_830001	50 S ribosomal protein L7/L12	N	N	−1,7	0,03	−1,1	0,03
RpsT	BC_4320		NP_834032	30 S ribosomal protein S20	N	N	ND	ND	−2	0,03
RplX	BC_0142		NP_830022	50 S ribosomal protein L24	N	N	−2,2	0,03	−0,79	0,154
RpmD	BC_0149		NP_830029	50 S ribosomal protein L30	N	N	−1,03	0,095	−2,4	0,02
Frr	BC_3822		NP_833543	Ribosome recycling factor	N	N	−1,6	0,04	−0,23	0,405
RaiA	BC_5190		NP_834854	SSU ribosomal protein S30P	N	N	−2,3	0,04	−2,6	0,02
EftS	BC_3824		NP_833545	Elongation factor Ts	N	N	1,263	0,204	−3,1	0,01
Transcriptional Regulation										
AbrB	BC_0042		NP_829939	Transcription state regulator	N	N	−1,15	0,082	−3,3	0,00
CodY	BC_3826		NP_833547	Transcriptional repressor	N	N	ND	ND	−2,6	0,02
-	BC_3728		NP_833453	DNA-binding protein HU	N	N	−0,23	0,422	−2,5	0,00
Other										
-	BC_1984		NP_831667	Phage protein	N	N	3,113	0,134	4,77	0,01
-	BC_1012		NP_830798	unknown	N	N	−0,81	0,291	−3,5	0,00
-	BC_5360		NP_835021	unknown	Y	Y	0,299	0,134	2,38	0,00
-	BC_0002		NP_829890	unknown	N	N	−4,1	0,00	−3,1	0,01
-	BC_2077		NP_831845	unknown	N	N	−4,6	0,00	−0,01	0,477
-	BC_4062		NP_833780	unknown	Y	N	0,31	0,363	0,9	0,01
-	BC_2705		NP_832458	unknown	N	N	−1,89	0,084	−3,6	0,00

Only changes satisfying statistical criteria (*p*-value<0.05) at least in one growth condition are shown.

a Each log_2_ fold-change value represents the mean protein level of the Δ*rex* sample in relation to the wild-type sample. The relative amount of each protein was determined using PatternLab software. Plus and minus symbols indicate up-and down regulation of the protein, respectively. Numbers in bold indicate data that satisfied statistical criteria (*p*-value<0.05). ND: not detected. For details, see Table S7.

#### (iii) Predicted cytoplasmic proteins

**Figure 4:**
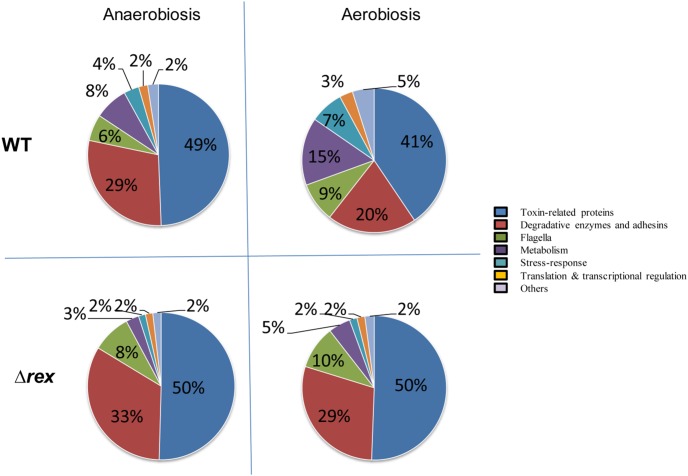
Functional distribution of proteins identified in Δ*rex* and wild-type *B. cereus* exoproteomes under aerobiosis and anaerobiosis. The diagrams represent the average proportion of each functional protein group based on total spectral counts (Table S5). For readability reasons, standard deviations (below 10%) are not shown.

In contrast to proteins predicted to be classically secreted, many proteins predicted to be cytoplasmic proteins, including metabolic proteins, stress response-related proteins, translation-related proteins, and other proteins were significantly less abundant in the exoproteome of the Δ*rex* mutant than the parental strain (Table 4). We noted that most of the cytoplasmic proteins that showed a significant abundance level decrease under aerobiosis did not show significant abundance level changes under anaerobiosis, and vice-versa (Table 4 and Table S7). Finally, the percentage of predicted cytoplasmic proteins significantly decreased in the Δ*rex* exoproteome compared with the wild-type exoproteome in the presence of oxygen, while the percentage of typically secreted proteins (toxin-related proteins and degradative enzymes and adhesins) was significantly higher (Fig. 4).

### The absence of Rex does not affect the mRNA level of toxin-related proteins

An explanation for the increased abundance levels of toxin-related proteins in the Δ*rex* mutant exoproteome could be a change in their mRNA levels. We thus tested whether transcription of genes encoding these proteins was altered in the Δ*rex* mutant compared with wild-type. Table 3 indicates that *rex* deletion significantly down-regulated *entC* and *entFM* under aerobiosis, but did not significantly change transcription of all other toxin-related genes. These transcriptomic results are not concordant with the proteomic data. This suggests that toxin-related protein abundance may be mainly modulated at the post-transcriptional level when Rex is lacking.

## Discussion

*B. cereus* Rex is an authentic Rex transcriptional factor because (i) it is able to bind to promoter fragments *in* *vitro*, indicating that at least in some cases the effect of Rex is mediated by direct interaction with the promoters, (ii) it impacts the cellular metabolism and oxidative stress tolerance, and (iii) it acts mainly as a repressor at the proteomic level [12], [13], [14], [15], [20], [48]. However, *B. cereus* Rex also plays an important role in fully aerobic respiratory metabolism and it modulates the toxinogenic profile of exoproteome.

When oxygen was supplied to pH-regulated batch cultures at pO_2_ = 100%, *B. cereus* metabolizes glucose to carbon dioxide by oxidation of glycolytic pyruvate in the tricarboxylic acid (TCA) cycle. This reaction produces NADH, which then fuels oxidative phosphorylation to produce ATP, with minimal production of lactate and elimination of carbon excess through excretion of acetate. Rex deficiency facilitates the entry of carbon flow into the NADH-recycling lactate pathway at the expense of pyruvate oxidation into acetyl-CoA (Fig. 1). This promotes lactate production at the expense of acetate without impairment of TCA capacity, since acetate was always produced as a waste [28]. In the absence of oxygen or other external electron acceptors, ATP synthesis occurs at the level of substrate phosphorylation and NADH is reoxidized in terminal step fermentative reactions from pyruvate (Fig. 1). When grown in pH-controlled anaerobic batch cultures (pO_2_ = 0%), *B. cereus* produces lactate as the main glucose by-product to satisfy the demand for redox balance. Rex deficiency favored the production of more reduced metabolites, as evidenced by the increase in the ethanol to acetate ratio and the decrease of lactate production. This indicates that Rex regulates the carbon flow distribution at the pyruvate node by favoring carbon flow through the NADH-recycling lactate pathway at the expense of Pfl-dependent fermentative pathways under anaerobiosis. A key question is how Rex controls the carbon flow into the NADH-recycling lactate pathway in *B. cereus* cells grown in fully aerobic respiratory and anaerobic fermentative conditions? This control process could involve interplay of transcriptional and/or post-transcriptional regulation of key glycolytic enzymes (such as Pgm, BC_4919 under anaerobiosis and PK, BC_4599 under aerobiosis) and post-translational regulation of lactate dehydrogenase. Evidently, further intensive work is required to unravel the complexity of the regulatory network involving Rex. Finally, by controlling the entry of carbon flow into the lactate pathway, Rex controls the availability of glycolytic intermediates for macromolecular synthesis as well as supporting NADPH production under both aerobiosis and anaerobiosis.

In contrast to anaerobic fermentation, aerobic respiration is a major source of reactive oxygen species (ROS) generation [49]. *B. cereus*, like other facultative anaerobes, uses scavenging systems to control the level of ROS [50]. Sequestration of ROS leads to oxidative deactivation of these scavenging systems, which are then reactivated directly or indirectly depending on NADPH level [51]. By modulating the distribution pattern of antioxidant enzymes (Tables 2 and 4), and possibly NADPH production, Rex may thus modulate the effectiveness of antioxidative defense systems. Specifically, Rex may maximize antioxidant system activity under anaerobiosis while restricting this activity under aerobiosis; this could explain why the resistance of *B. cereus* cells towards external H_2_O_2_, when Rex is absent, is lower under anaerobiosis and higher under aerobiosis.

Except for HlyI, the abundance levels of classical extracellular proteins (such as toxins) were enhanced in *B. cereus* exoproteome when Rex was absent, especially under aerobiosis. In *B. cereus*, as in other Gram-positive bacteria, most extracellular proteins are synthesized as precursors, with typical N-terminal signal peptides, and exported from the cytoplasm by the Sec-dependent pathway [52]. This is the case for toxins [6]. However, unlike other toxins, HlyI contains a putative N-terminal twin arginine motif that could target the protein to the Tat secretion pathway instead of the Sec-dependent pathway [53], [54]. Besides classical export routes, *B. cereus* possesses specialized protein export systems such the flagellar type III system [5], [55], which translocates most flagellar components across the cytoplasmic membrane, and possibly the ESAT-6 secretion system, which mediates the secretion of virulence factors belonging to the WXG100 protein family, such as the protein BC_5360 [56], [57]. A feature of these two specialized secretion systems is the lack of a classical signal peptide to direct the substrate protein for secretion. However, like the Sec-dependent pathway and unlike the TAT-dependent pathway, these protein export systems require chaperones to prevent premature folding, aggregation and degradation by cytoplasmic proteases [58], [59]. Rex may thus influence protein-assisted export by modulating the distribution pattern of the components of export systems as observed in our proteomic data (Table 2, [60]). In addition, it has been reported that chaperones may be targets for intracellular ROS [61]. Keeping the functional integrity of these chaperones, specifically in the context of secretion, may thus depend on Rex-dependent NADPH availability. The role and contribution of NADPH in classical and specialized secretory mechanisms remains an open question that deserves further experiments.

Our results show that many cytoplasmic proteins predicted to be involved in active growth, either directly (such as metabolic enzymes, translational and post-translational-related proteins) or indirectly by preventing inappropriate gene expression (such as AbrB and CodY under aerobiosis [62]), were less abundant in *B. cereus* exoproteome when Rex was absent. We assume that cell lysis cannot account for changes in the abundance level of these cytoplasmic proteins since: (i) cell viability count did not change (data not shown), (ii) EF-Tu did not accumulate in extracellular medium (Table S7, [63]), and (iii) the abundance pattern of cytoplasmic proteins was dependent on growth conditions and did not always correlate with intracellular abundance changes (Table 2 and 4). Among the cytoplasmic proteins identified in *B. cereus* exoproteome, several have already been identified in the exoproteome of other bacteria, and many have been described as extracellular moonlighting components playing a role in bacterial virulence [64], [65]. The non classical mechanism of secretion that could explain their release remains to be firmly described [47], [66]. One hypothesis is that signal-less predicted cytoplasmic proteins might be secreted within outer membrane vesicles [63]. Such nanovesicles have been observed in *Bacillus anthracis* [67]. Interestingly, ATP is the primary metabolic factor involved in secretory vesicle movements. ATP generation is lower in fermenting cells than in non-fermenting cells, according to metabolism. In addition, Rex promotes ATP generation by enhancing NADH-producing pathways at the expense of NADPH pathways. Therefore, if ATP availability is a triggering factor for export of cytoplasmic proteins, this could explain the higher export of cytoplasmic proteins in aerobically grown cells compared to anaerobically grown cells, as well as the substantial decrease of cytoplasmic protein export under aerobiosis when Rex was absent.

In conclusion, Rex in *B. cereus* plays a pivotal role in controlling the cross-talk between the metabolic networks that control growth, oxidative defense machinery and extracellular accumulation of toxins. In the context of *B. cereus* natural environment, Rex may thus help the pathogen to (i) maintain its host alive until transmission to the next host can be achieved, by controlling its growth and production of its toxins [68], and (ii) better survive stress conditions under anaerobiosis, albeit to the detriment of maximizing its survival when oxygen is present [10], .

## Supporting Information

Figure S1 **Genetic organization of** ***rex*** **chromosomal region of** ***B. cereus*** **ATCC 14579. Panel A.** Large arrows represent open reading frames identified in strain ATCC 14579, and their orientation shows the transcriptional direction. **Panel** **B.** Nucleotide sequence of *rex* ORF (black capital letters), as well as promoter and terminator regions (blue letters), are shown. Transcriptional start site (+1), putative −35 and −10 motifs and putative terminator are underlined. Start and stop translation codons are in bold.
(TIF)

Figure S2 **Survival of** ***B. cereus*** **F4430/73 cells towards external hydrogen peroxide.** Cells were grown in liquid cultures to mid-exponential growth phase either aerobically or anaerobically, and subjected to 20 and 10 mM H_2_O_2_ stress, respectively. Samples were taken at 0 and 20 min. Colony forming units per mL were counted and expressed as (N/No)×100. Error bars represented the standard deviation from three independent measures. Significant differences (p<0.05) between mutant and wild-type strains are indicated with asterisks.
(TIF)

Figure S3 **SDS-PAGE analysis of overproduced and purified** ***B. cereus*** **Rex.** Rex purification fractions were analyzed by electrophoresis on an SDS-12% polyacrylamide gel after Coomassie Brillant Blue staining (Panel A) or Imperial Protein Stain (Thermo scientific, Panel B). Position and molecular weights (kDa) of markers (lanes 1) are given on the left. **Panel A**: Lane 1, standard proteins; Lane 2, soluble whole cell extract from *E. coli.* **Panel B**: Lane 1, standard proteins; Lane 2, purified protein after Co^2+^ IMAC.
(TIF)

Figure S4 **Venn diagrams summarizing proteins that showed significant abundance level changes in the cellular and extracellular proteomes of Δ** ***rex*** **compared with wild-type** ***B. cereus*** **cells under anaerobiosis (blue) and aerobiosis (red).** Proteins up- and down-regulated by Δ*rex* were distinguished.
(TIF)

Table S1 **Results from controlled batch cultures of Δ** ***rex*** **mutants and its parent strain,** ***B. cereu*** **s F4430/73.**
(DOCX)

Table S2 **List of assigned MS/MS spectra from the cellular proteomes.**
(XLSX)

Table S3 **List of assigned MS/MS spectra from the exoproteomes.**
(XLSX)

Table S4 **List of proteins identified in cellular proteomes and their normalized spectral count.**
(XLSX)

Table S5 **List of proteins identified in exoproteomes and their normalized spectral count.**
(XLSX)

Table S6 **Fold changes of cellular proteins and their statistical significance.**
(XLSX)

Table S7 **Fold changes of exoproteins and their statistical significance.**
(XLSX)
