# Supplementary figures and images for: Proteomic Evidences for Rex Regulation of Metabolism in Toxin-Producing Bacillus cereus ATCC 14579
Source: PLoS One. 2014 Sep 12;9(9):e107354. doi: 10.1371/journal.pone.0107354 (PMC4162614; doi:10.1371/journal.pone.0107354)

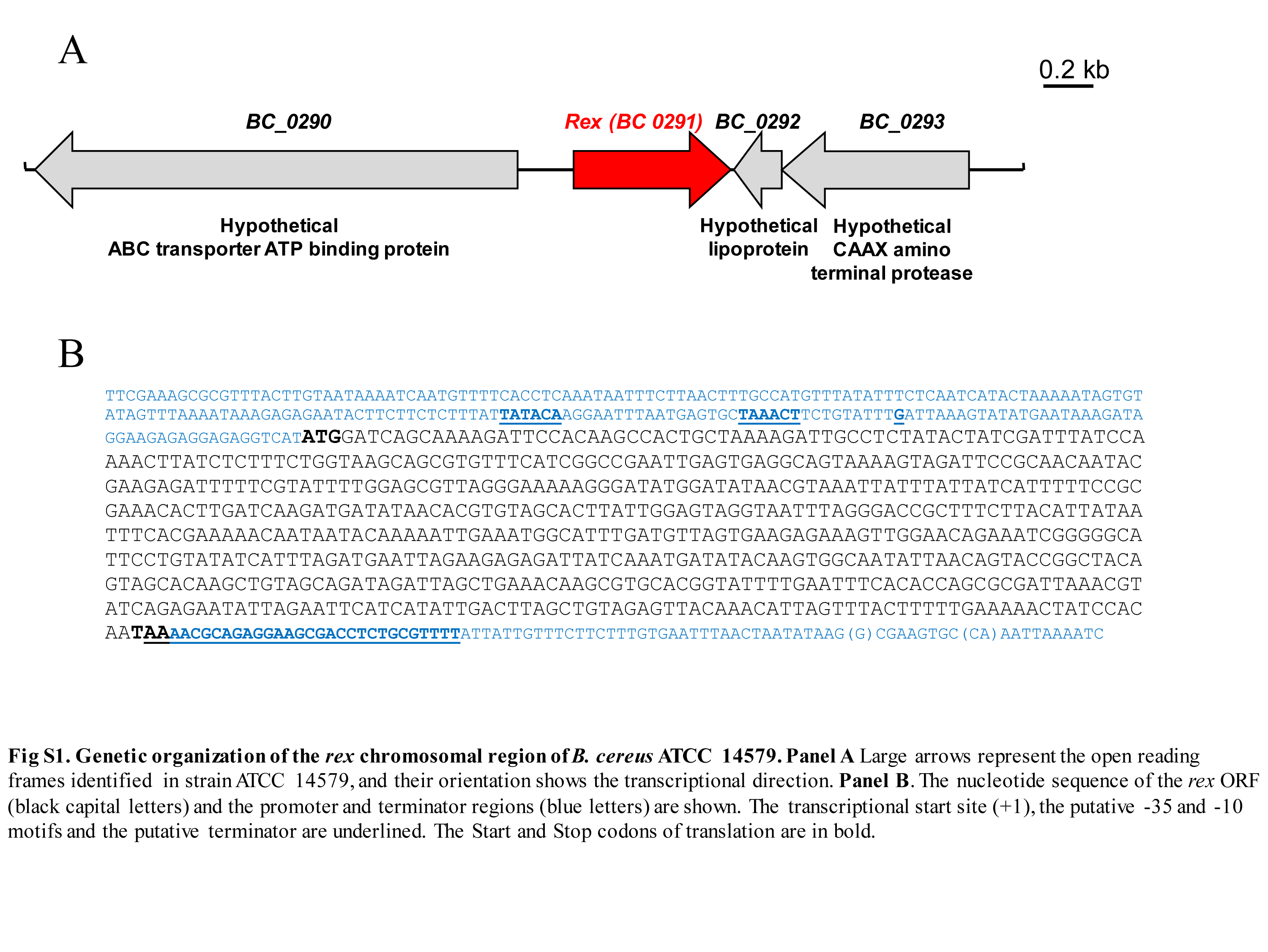

Supplement: Figure S1 — Genetic organization of rex chromosomal region of B. cereus ATCC 14579. Panel A. Large arrows represent open reading frames identified in strain ATCC 14579, and their orientation shows the transcriptional direction. Panel B. Nucleotide sequence of rex ORF (black capital letters), as well as promoter and terminator regions (blue letters), are shown. Transcriptional start site (+1), putative −35 and −10 motifs and putative terminator are underlined. Start and stop translation codons are in bold. (TIF) [file pone.0107354.s001.tif]

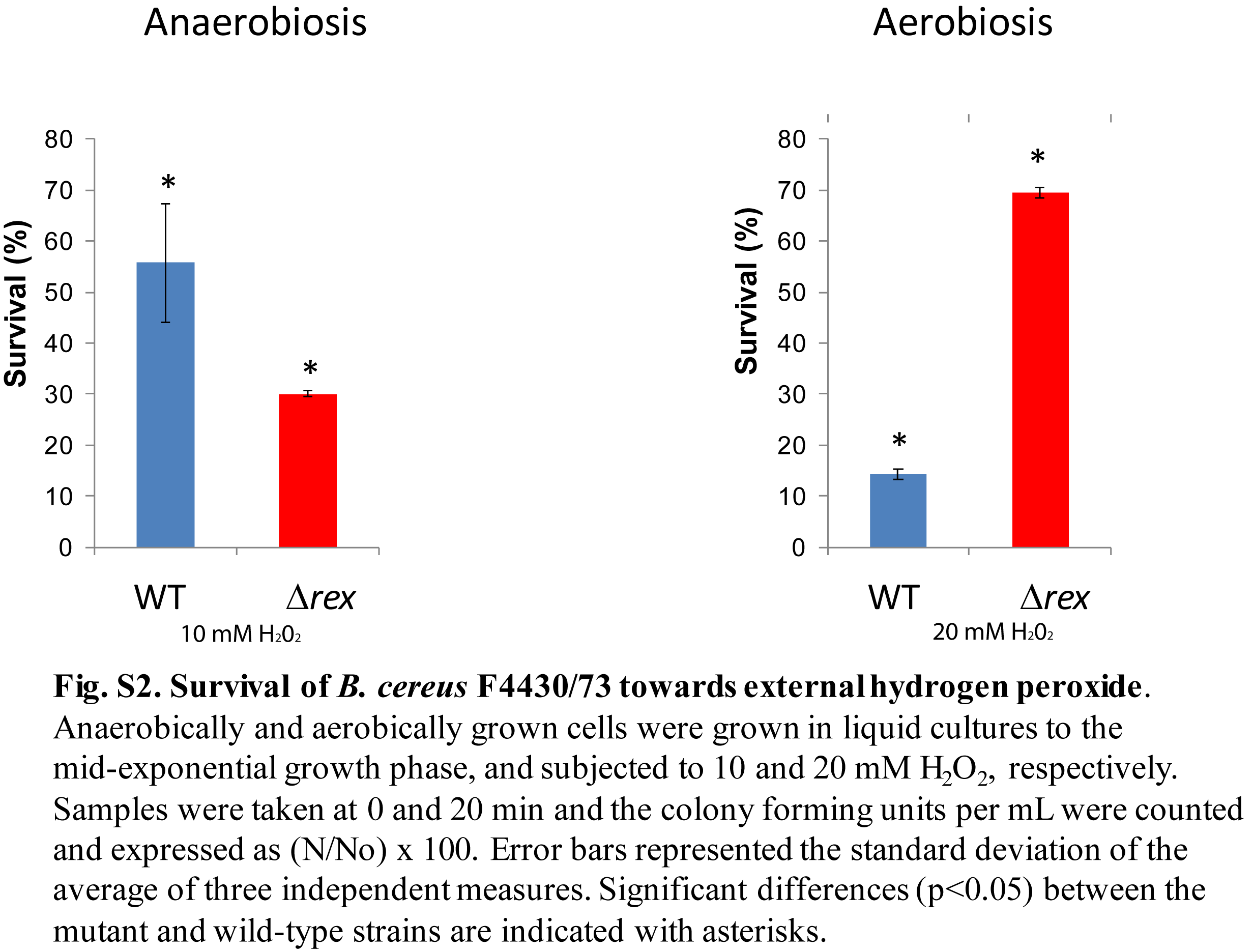

Supplement: Figure S2 — Survival of B. cereus F4430/73 cells towards external hydrogen peroxide. Cells were grown in liquid cultures to mid-exponential growth phase either aerobically or anaerobically, and subjected to 20 and 10 mM H2O2 stress, respectively. Samples were taken at 0 and 20 min. Colony forming units per mL were counted and expressed as (N/No)×100. Error bars represented the standard deviation from three independent measures. Significant differences (p<0.05) between mutant and wild-type strains are indicated with asterisks. (TIF) [file pone.0107354.s002.tif]

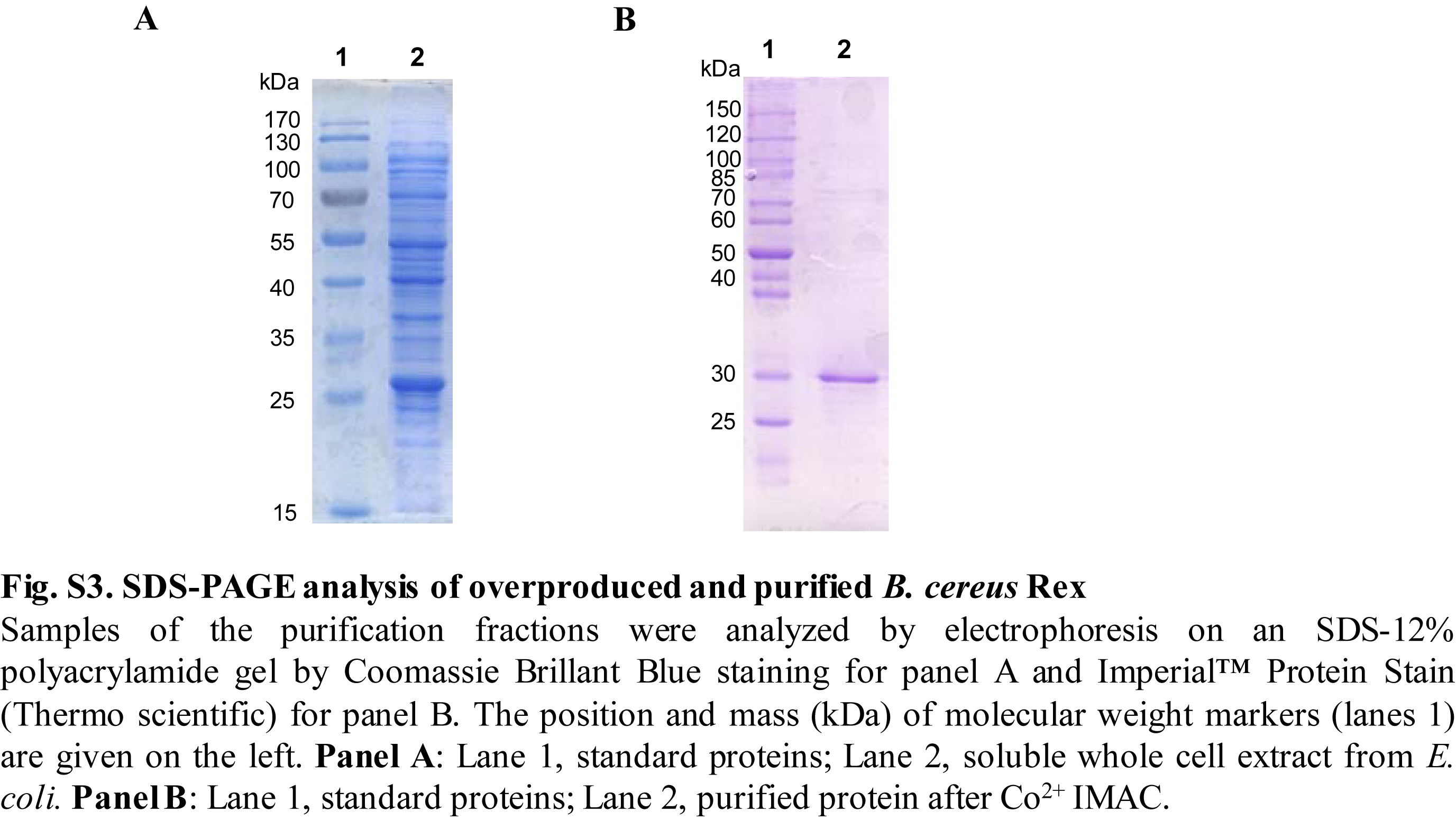

Supplement: Figure S3 — SDS-PAGE analysis of overproduced and purified B. cereus Rex. Rex purification fractions were analyzed by electrophoresis on an SDS-12% polyacrylamide gel after Coomassie Brillant Blue staining (Panel A) or Imperial Protein Stain (Thermo scientific, Panel B). Position and molecular weights (kDa) of markers (lanes 1) are given on the left. Panel A: Lane 1, standard proteins; Lane 2, soluble whole cell extract from E. coli. Panel B: Lane 1, standard proteins; Lane 2, purified protein after Co2+ IMAC. (TIF) [file pone.0107354.s003.tif]

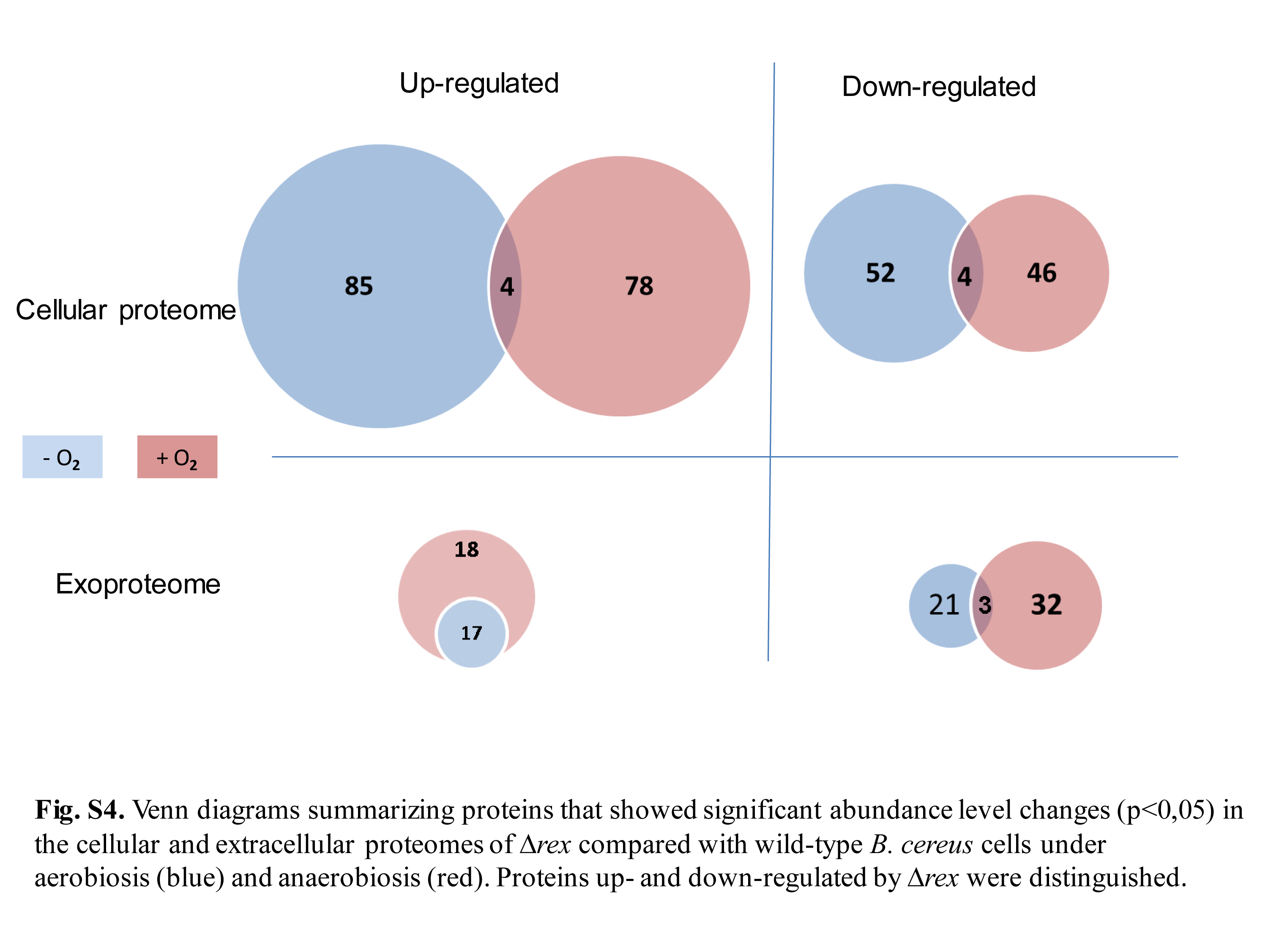

Supplement: Figure S4 — Venn diagrams summarizing proteins that showed significant abundance level changes in the cellular and extracellular proteomes of Δ rex compared with wild-type B. cereus cells under anaerobiosis (blue) and aerobiosis (red). Proteins up- and down-regulated by Δrex were distinguished. (TIF) [file pone.0107354.s004.tif]
