# Supplementary material for: Proteomic Evidences for Rex Regulation of Metabolism in Toxin-Producing Bacillus cereus ATCC 14579
Source: PLoS One. 2014 Sep 12;9(9):e107354. doi: 10.1371/journal.pone.0107354 (PMC4162614; doi:10.1371/journal.pone.0107354)
Supplement: Table S1 — Results from controlled batch cultures of Δ rex mutants and its parent strain, B. cereu s F4430/73. (DOCX) [file pone.0107354.s005.docx]

**Table S1.** Results from controlled batch cultures of Δ*rex* mutant and its parent strain,

*B. cereu*s F4430/73 ^a^.

|  | Anaerobic fermentative growth | |  |  | Aerobic respiratory growth | |
| --- | --- | --- | --- | --- | --- | --- |
|  | WT | Δ*rex* |  |  | WT^b^ | Δ*rex* |
| Maximal specific growth rate (µ_max_) (h^-1^) | 0.86± 0.04 | 0.92 ± 0.02* |  |  | 1.47 ± 0.01 | 1.69 ± 0.18* |
| Final biomass (g.liter^-1^) | 0.77 ± 0.01 | 0.88 ± 0.01* |  |  | 2.32 ± 0.17 | 2.15 ± 0.05 |
| *Y_glucose_* (g of cells. mol of glucose^-1^) | 26 ± 1 | 29 ± 1 |  |  | 77 ± 1 | 72 ± 1 |
| Maximal specific glucose consumption(mmol.g^-1^.h^-1^) | 33 ± 2 | 32 ± 2 |  |  | 19 ± 2 | 23 ± 2 |
| Yields of end products (mol.mol glucose^-1^)^c^ |  |  |  |  |  |  |
| Lactate (*Y*_l_*_actate_*) | 1.45 ± 0.01 | 1.23 ± 0.10* |  |  | 0.03 ± 0.01 | 0.23 ± 0.01* |
| Acetate (*Y_acetate_*) | 0.29 ± 0.01 | 0.44 ± 0.01* |  |  | 1.00 ± 0.01 | 0.80 ± 0.41 |
| Formate (*Y_formate_*) | 0.38 ± 0.01 | 0.73 ± 0.01* |  |  | NZ^d^ | NZ |
| Ethanol (*Y_ethanol_*) | 0.14 ± 0.01 | 0.27 ± 0.01* |  |  | NZ | NZ |
| Succinate (*Y_succinate_*) | 0.02 ± 0.01 | 0.02 ± 0.01 |  |  | NZ | NZ |
| Ethanol versus Acetate | 0.48 | 0.61* |  |  |  |  |
| ATP yield^e^ | 2.17 | 2.38 |  |  | ND | ND |
| NADH recovered^g^ | 1.2 | 1.1 |  |  | ND | ND |

^a^Cells were grown under N_2_- generated anaerobiosis (pO_2_ = 0%) and full aerobiosis (pO_2_ = 100%). Data are the means of triplicate measures obtained from three independent cultures.

^b^WT, wild-type parent strain F4430/73.

^c^Yields of end products were calculated at the stationary phase.

^d^NZ, yield was below 0.01 mol.mol glucose^-1^.

^e^ATP yield was calculated as moles of ATP produced per mole of consumed glucose, and was equal to *Y*_l_*_actate +_Y_ethanol_* + 2**Y_acetate_.*

^f^ND, not determined.

^g^NADH recovery was calculated as the ratio of pathways producing NADH versus those consuming NADH (producing NAD^+^), and was equal to (lactate + 2 x acetate + 2 x ethanol - formate)/(lactate + 2 x ethanol).

*p<0.05 vs WT in Student’s t-test.
